# Leiomyosarcoma of the base of the tongue and free edge of the epiglottis: a case report

**DOI:** 10.1186/1752-1947-6-400

**Published:** 2012-11-23

**Authors:** Adelchi Croce, Antonio Moretti, Melissa Laus, Domenico Crescenzi

**Affiliations:** 1 ENT Department, University ‘G. D’Annunzio’ of Chieti-Pescara, Hospital ‘SS. Annunziata’, Via dei Vestini, Chieti, 66100, Italy

**Keywords:** Base of the tongue, Free edge of the epiglottis, Leiomyosarcoma, Surgery

## Abstract

**Introduction:**

We present the case of a man with a leiomyosarcoma of the base of the tongue. We feel this case is important as this kind of pathology, though rare, can occur at a site where carcinomas are more frequent.

**Case presentation:**

A 77-year-old Caucasian man had been reporting difficulty in swallowing and hoarseness for a month before admission to our department. After several preliminary tests, including a biopsy which was positive for a malignant epithelial neoplasm which required further immunohistochemical study, we decided to operate, removing the base of our patient’s tongue and performing a total laryngectomy. Histological examination of the specimen revealed a high-grade leiomyosarcoma of the base of the tongue and of the free edge of the epiglottis.

**Conclusions:**

We wish to stress the rarity of this clinical case, related to the site of implantation of the tumor, as confirmed by the difficulties in finding reference to this topic in the international literature. In fact, several cases of leiomyosarcoma have been described, but in different locations from that seen in our patient’s case.

## Introduction

Soft tissue sarcomas account for approximately 0.7 percent of all malignant neoplasms, and leiomyosarcomas have been reported to account for 3 percent to 7 percent of soft tissue sarcomas [1,2]. The most frequent sites of occurrence are the uterine myometrium, gastrointestinal tract and skin [3]. The highest incidence occurs between 40 and 49 years of age. In fact, its presentation is unusual in children or in older patients. Women are more affected than men because of the uterine association. Leiomyosarcomas are infrequent in the oral cavity and oropharynx, but when present they are usually localized on the tongue, lips and palate [4]. The prognosis is poor, with a high percentage of recurrence or metastasis. The most common sites of metastasis include the lungs, bone, brain and the lymph nodes. This neoplasm is an extremely rare mesenchymal lesion in the oral cavity and oropharynx, with unusual bone location because of the paucity of smooth muscle in that site [5,6]. When it occurs in these locations, it appears as a slow-growth mass, symptomatic only in exceptional cases [7]. The main symptoms are pain, teeth mobility, or even difficulty in chewing, usually when the tumor is located in the tongue, lips or palate [8]. Due to its unspecific clinical presentation, diagnosis is made after histological study [9], where the typical small smooth muscle cells are seen, being of uniform size and with no features of malignancy. In order to achieve more specific analysis and more precise differential diagnosis immunohistochemical studies must be carried out. Surgery is the only effective treatment at this time. It is important to perform a complete resection in order to avoid recurrences [8]. This is usually easily achieved due to its characteristics as a well-circumscribed tumor. Here, we describe a case of leiomyosarcoma of the base of the tongue. From a histological point of view this is a rare kind of pathology in a site where carcinomas are more frequent.

## Case presentation

We present the case of a 77-year-old Caucasian man with a leiomyosarcoma of the base of the tongue. He had reported difficulty in swallowing and hoarseness for a month before admission to our department. He also reported a history of dyspnea during exercise and chronic obstructive pulmonary disease (COPD) treated with salmeterol 50μg (two puffs/day). He was a long-term smoker, with lipid disorders and allergy to aspirin.

**Figure 1:**
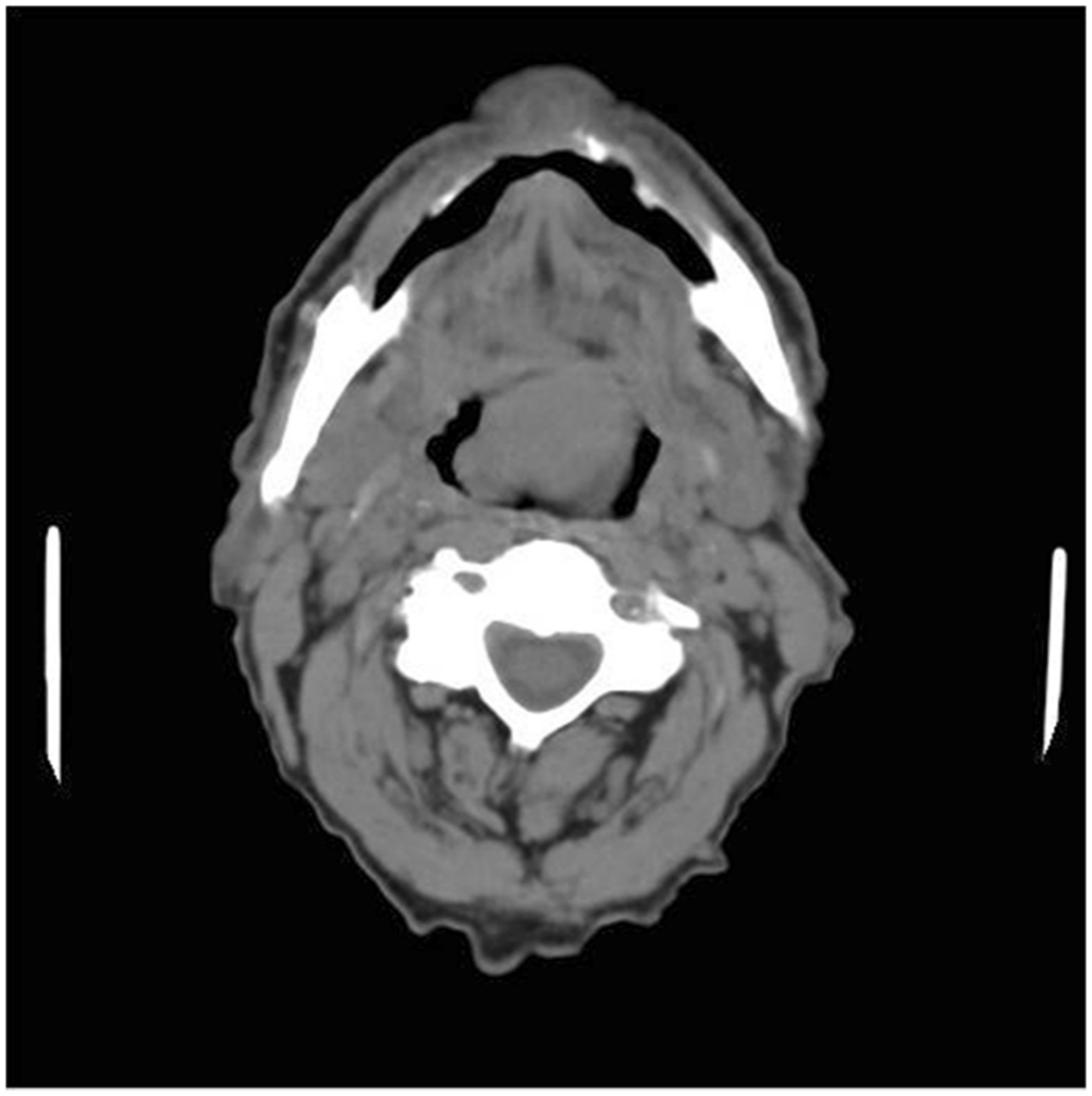
Computed tomography image demonstrating the mass.

Endoscopy and a computed tomography scan showed a mass on the basis of the tongue (Figure 1), on which a biopsy was performed. The biopsy results indicated a malignant epithelial neoplasm, which required further immunohistochemical studies, ulcerated and infiltrative with spindle cells on the base of the tongue and free edge of the epiglottis.

Our patient was hospitalized and underwent surgery. Before the operation, we performed a percutaneous endoscopic gastrostomy (PEG) procedure to facilitate the nutrition of our patient after the intervention, since removal of the tongue base can be associated with difficulties in swallowing.

After insertion of a central venous line in the right subclavian vein our patient underwent surgery consisting of a tracheostomy performed under general anesthesia, preparation of a cervical flap, bilateral neck dissections (levels 2 to 4) on which an extemporaneous examination was performed (proving negative for lymph node metastases), isolation of the hypoglossal nerves and examination of their entry into the tongue base to preserve these structures, total laryngectomy extended to the base of the tongue, preparation of a myofascial flap of the left pectoralis major muscle (chosen because our patient is right-handed) and its positioning at the base of the tongue, placement of drainages and trachea tube and dressing.

**Figure 2:**
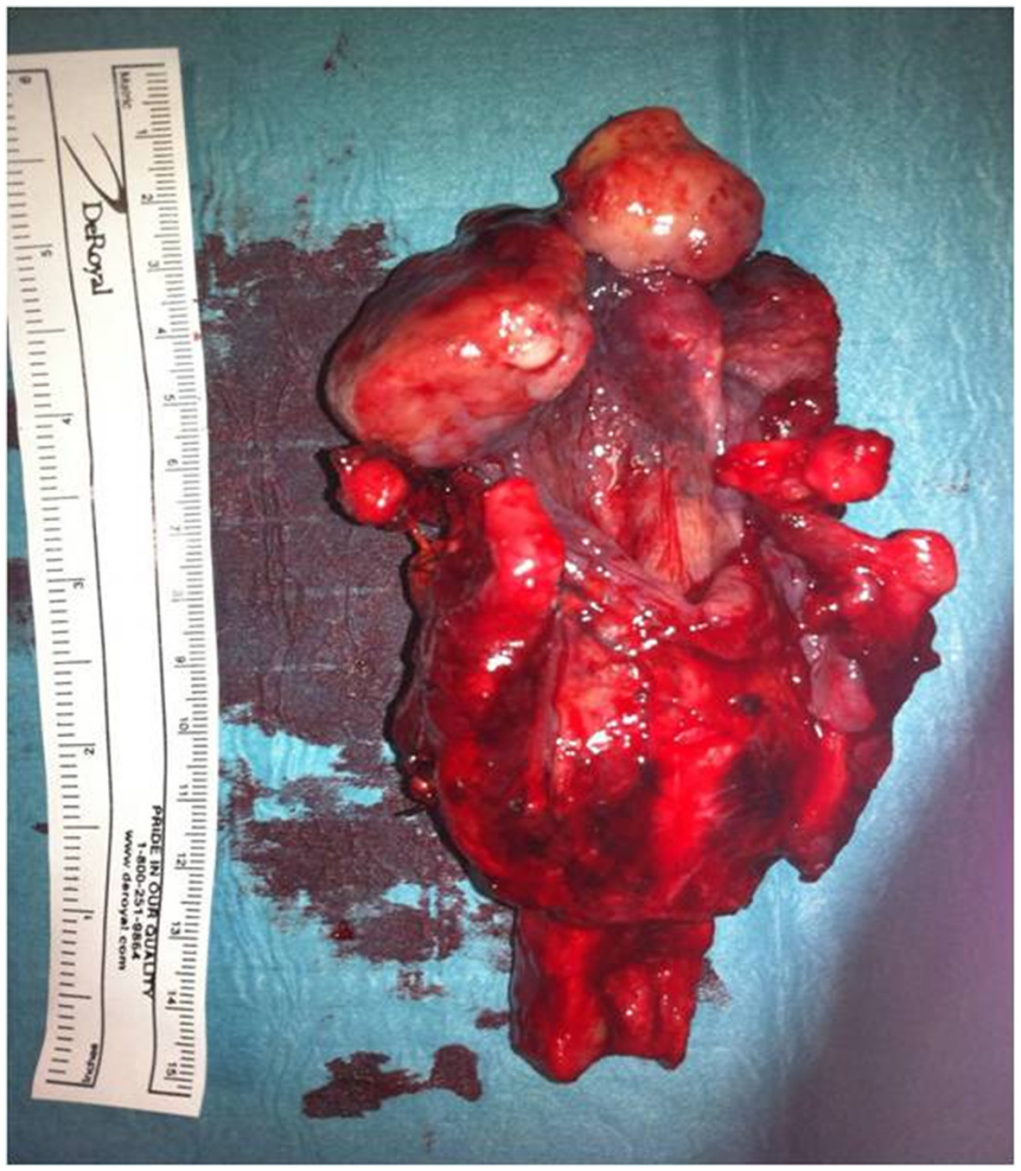
Posterior macroscopic view of the surgical specimen.

The definitive histological examination performed on surgical specimens (Figure 2) showed leiomyosarcoma of the base of the tongue and free edge of the epiglottis and the absence of neoplastic infiltration of surgical resection margins. Immunohistochemical studies performed on the biopsy confirmed the same diagnosis.

Our patient had a regular post-operative course and was discharged from our clinic, able to use both PEG enteral and mouth feeding.

To date, about eight months after surgery, our patient remains disease free.

## Discussion

Leiomyosarcoma is a malignant neoplasm that originates from smooth muscle. It frequently occurs in the uterine myometrium, gastrointestinal tract, retroperitoneum, skin and subcutaneous tissue. It rarely occurs in the head and neck, probably because of the paucity of smooth muscle tissue in these sites [5]. The most common sites of leiomyosarcoma in the oral cavity and oropharynx are the tunica media of blood vessels, the ductus lingualis, the circumvallate papillae, the myoepithelial cells or the undifferentiated mesenchymal cells. Other locations include the lips, tongue, and hard and soft palate. The most common sites of leiomyosarcomas include the maxilla and mandible [10].

**Figure 3:**
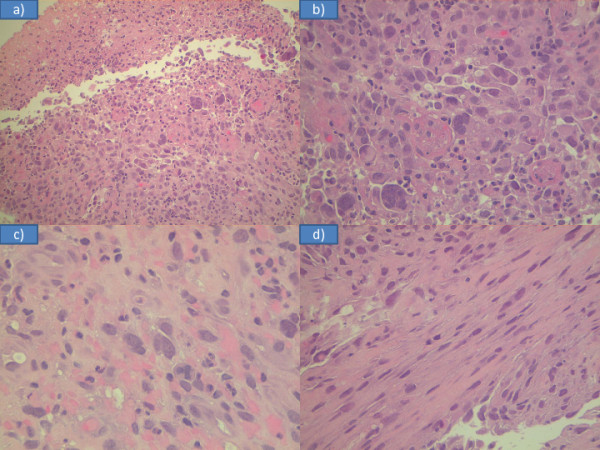
Histological examination of biopsy: (a) necrosis and atypia of the tumor; (b) plurinucleate cells; (c) cell with atypical mitosis;(d) fusiform aspects.

**Figure 4:**
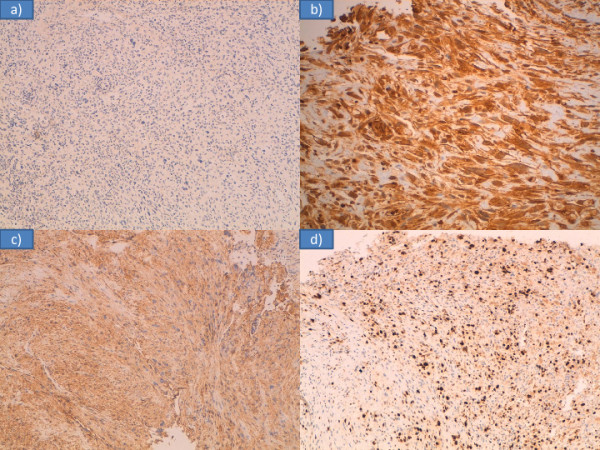
Immunohistochemical studies: (a) cytokines AE1 and AE2 negative; (b) vimentin positive; (c) smooth muscle actin positive, (d) proliferation index ‘MIB1’ was 60 percent.

**Figure 5:**
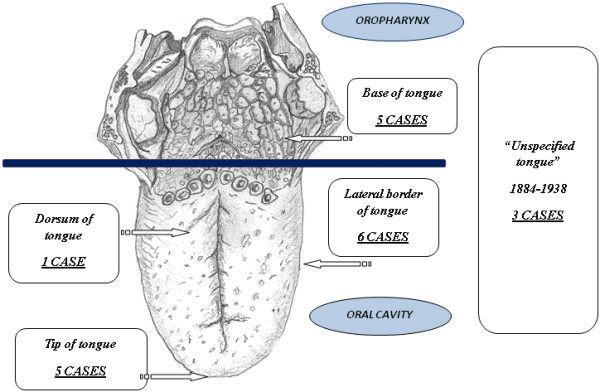
**Locations of leiomyosarcomas as reported in the literature (see also Table**1**).**

Primary leiomyosarcoma of the tongue is a rare lesion. The cause of leiomyosarcoma remains unknown, although cases have been associated with trauma, estrogenic stimulation, ionizing irradiation and Epstein-Barr virus [11,12]. Diagnosis of leiomyosarcoma is based on pathologic criteria. The histological criteria are based on a typical pattern of interlacing bundles of smooth muscle cells with a high mitotic rate, pleomorphism, and bizarre cell forms; these are all indicative of malignancy. The differential diagnosis includes several types of tumor characterized by prominent spindle cell features and may be extremely difficult when these tumors display atypical morpho-architectural features and/or arise in uncommon sites. Immunohistochemistry or electron microscopy are accepted as useful tools for confirming the diagnosis [13-15]. In our patient’s case it was not possible to make a definitive diagnosis on biopsy. Initially atypical cells, atypical mitoses and fusiform aspects led us to a diagnosis of poorly differentiated epidermoid carcinoma with spindle cell aspects (Figure 3). Combining morphological and immunohistochemical studies we came to the final diagnosis. In fact, test results for cytokines AE1 and AE2 were negative (excluding the diagnosis of carcinoma), vimentin and smooth muscle actin were positive (confirming the sarcomatous nature of the lesion), and the proliferation index ‘MIB1’ was quantified to 60 percent (Figure 4). To date, we only know of 20 cases reported (including our patient’s case) of primary leiomyosarcoma of the tongue (Table 1) (Figure 5) [15-33].

**Table 1:** Previously recorded cases of leiomyosarcoma of the oral cavity and oropharynx

**Authors**	**Date**	**Sex**	**Age**	**Location**	**Treatment**
Blanc *et al. Gaz Hebd Med Chir* 21: 611 [16]	1884	M	33	Tongue	-
Glas *et al. Wien Klin Wscher* 18:168 [17]	1905	M	44	Tongue	-
Stout *et al. Am J Cancer* 34:31 (case 1) [18]	1938	M	50	Tongue	-
Stout *et al. Am J Cancer* 34:31 (case 2) [18]	1938	W	29	Base of tongue	-
Burford *et al. Am J Orthhod* 30:395 [19]	1944	M	32	Base of tongue	-
Yannopoulo *et al. Cancer* 15: 958 [20]	1962	M	1	Tip of tongue	Excision
Bertelli *et al. Oral Surg* 19: 771 [21]	1965	M	43	Tip of tongue	-
MacDonald *et al. Br J Oral Surg* 6:207 [22]	1969	W	11	Dorsum of tongue	Excision
Goldberg *et al. J Oral Surg* 28:608 [23]	1970	M	54	Tip of tongue	Excision
Lack *et al. Pediatr Pathol* 6:181 [24]	1986	M	2.5	Base of tongue	Excision and chemiotherapy
Aydin *et al. Oral Oncol Eur F Cancer* 30B(5):351 [25]	1994	M	70	Base of tongue	Radiation therapy
Mayall *et al. J Laryngol Otol* 108:617 [26]	1994	M	60	Tip of tongue	Excision
Piattelli *et al. J Oral Maxil Surg* 53 M:698 [27]	1995	W	80	Lateral border of tongue	Patient rejected treatment
Lo Muzio *et al. Oral Oncol* 36:519 [28]	2000	M	67	Lateral border of tongue	Excision
Vora *et al. Oto H N Surg* 128:601 [29]	2003	W	62	Lateral border of tongue	Excision
Yang *et al. Int J Oral Maxil Surg* 35:469 [30]	2006	W	54	Tip of tongue	Excision
Castaldi *et al. Acta Radiol* 47:514 [31]	2006	W	52	Lateral border of tongue	Excision
Crossman *et al. Br J Oral Maxil Surg* 46:e69 [32]	2008	W	46	Lateral border of tongue	Excision
Pires *et al. Med Oral Pathol Oral Radiol Edod* 109:e31 [33]	2010	M	55	Lateral border of tongue	Excision
Present work	2011	M	77	Base of tongue	Excision

Table 1 provides a general overview on this topic and leads to a number of observations.

The age of these patients ranged from one to 80 years with a peak between the fifth or sixth decade of life, and there seems to be a slight gender predilection for men as seen in our experience. The sites of origin of the tumor lesions in the tongue include the tip, the lateral border and the base (Figure 5). It is interesting to note that our patient is one of the oldest of the five patients described with sarcoma of the base of the tongue.

The main treatment is excision with sufficient tumor free borders and post-operative irradiation when necessary. Radical neck dissection is reserved for cases with regional node involvement. Chemotherapy is generally reserved for palliative care and occasionally improves the survival time and quality of life of patients with metastatic disease or inoperable tumors [8].

The prognosis of oral leiomyosarcoma affecting the tongue is good if clear excision can be achieved.

In our patient’s case, we decided to treat the tumor surgically by removing the base of the tongue after planning at the beginning of treatment the preparation and placement of a myofascial flap of major pectoralis muscle to the remaining tongue in order to limit post-intervention swallowing problems from loss of the muscle tissue involved in swallowing. In the same way, in the light of possible post-operative complications, total laryngectomy was performed together with the removal of the base of the tongue due to the high risk of aspiration related to swallowing problems after partial laryngectomy in our patient with chronic obstructive pulmonary disease (COPD).

Our decision to remove the bilateral laterocervical lymph nodes was dictated by the response of the biopsy, which did not indicate sarcoma, but carcinoma; the high risk of bilateral lymph nodes metastases from base of the tongue carcinomas is well known.

In addition, after a multidisciplinary consultation we decided not to subject our patient to radiotherapy; this was in light of observation of the negative margins of the surgical specimen, and respecting the wishes of our patient. This decision could possibly be questionable, given the tendency to local recurrence of sarcoma.

## Conclusions

The number of reported cases of this malignant lesion of the tongue remains extremely small, but it is interesting to underline that our patient is the oldest man among the cases reported and his base of the tongue tumor was treated surgically.

Our surgical choices were based on the characteristics of lesions (especially their location), but also on the overall health of our patient.

Further data about these rare cases are needed to better understand their features in the future.

## Consent

Written informed consent was obtained from the patient for publication of this case report and any accompanying images. A copy of the written consent is available if required by the Editor-in-Chief of this journal.

## Competing interests

The authors declare that they have no competing interests.

## Authors’ contributions

All authors contributed to the manuscript writing. All authors read and approved the final manuscript.
